# Editorial: Multiple Herbicide-Resistant Weeds and Non-target Site Resistance Mechanisms: A Global Challenge for Food Production

**DOI:** 10.3389/fpls.2021.763212

**Published:** 2021-10-28

**Authors:** Joel Torra, María Dolores Osuna, Aldo Merotto, Martin Vila-Aiub

**Affiliations:** ^1^Department d'Hortofructicultura, Botànica i Jardineria, Agrotecnio-CERCA Center, Universitat de Lleida, Lleida, Spain; ^2^Center for Scientific and Technological Research of Extremadura (CICYTEX), Agrarian Research Center “Finca La Orden” Valdesequera, Badajoz, Spain; ^3^Department of Crop Science, Faculty of Agronomy, Federal University of Rio Grande Do Sul (UFRGS), Porto Alegre, Brazil; ^4^Department of Ecology, IFEVA -CONICET, Faculty of Agronomy, University of Buenos Aires (UBA), Buenos Aires, Argentina

**Keywords:** cross-resistance, cytochrome P450 monooxygenase (CYP450), enhanced herbicide metabolism, glutathione-S-transferase (GST), glyphosate, rapid necrosis

## Evolution of Multiple Survival Mechanisms

The acquired inheritable trait of plants to survive and reproduce under herbicide exposure is defined as resistance. Herbicide resistance is an extraordinary example of adaptive evolution in weed species infesting agroecosystems with clear detrimental consequences on agriculture sustainability around the globe (Palumbi, 2001; Llewellyn et al., 2016). Multiple herbicide resistance is a compelling evolutionary process in which distinct survival mechanisms are present in a population or are combined within single plants, each endowing resistance to dissimilar site of action herbicides (Hall et al., 1994; Gaines et al., 2020). These multiple mechanisms may involve either target site (TSR) or non-target site resistance (NTSR) mechanisms or any combination endowing multiple resistance. Multiple resistance can evolve through unique events that sequentially select for resistance alleles within single plants and/or genetic exchange of independently evolved resistance mutations through pollen outcrossing among plants within or between populations. Regardless of the driving factor, the ultimate result is the stack of various distinct survival mechanisms at the plant and/or population level endowing broad resistance to multiple herbicides of dissimilar chemistries.

Genetic variability and reproductive biology of weed species are the most important factors that define the likelihood of multiple resistance evolution. *Lolium rigidum, Alopecurus myosuroides, Raphanus raphanistrum*, and *Amaranthus spp*. are among the weed species with the most remarkable ability to evolve multiple resistance through eco-evolution of TSR and NTSR mechanisms (Hall et al., 1997; Cocker et al., 1999; Walsh et al., 2004; Owen et al., 2014, 2015; Schultz et al., 2015; Han et al., 2016; Tétard-Jones et al., 2018). For instance, resistance due to reduced glyphosate and paraquat translocation co-evolving with an *ACCase* target site mutation has been identified in a single *L. rigidum* population (Yu et al., 2007), whereas other patterns of multiple resistance in this species reflect the presence of enhanced CYP-450 herbicide metabolism coexisting with *ACCase, ALS*, α*-tubulin*, and/or *EPSPS* point mutations (Burnet et al., 1994a,b; Tardif and Powles, 1994; Neve et al., 2004; Han et al., 2016, 2021; Chen et al., 2018, 2020a). Another striking example of multiple resistance is found in *A. tuberculatus* populations where *PPO, ALS*, and *EPSPS* target site mutations have been identified co-evolving with enhanced metabolism of PSII and HPPD inhibiting herbicides (Schultz et al., 2015).

Novel resistance mechanisms in weeds have been identified recently although some were thought unlikely to evolve. For instance, glyphosate resistance is possible through aldoketoreductase (AKR)-based metabolism (Pan et al., 2019), up-regulation of an ABC membrane transporter pumping out glyphosate outside the cell (Pan et al., 2021) and programmed cell death causing rapid necrosis (Van Horn et al., 2018). Likewise, 2,4-D resistance due to either CYP-450 based metabolism (Giacomini et al., 2020), a double point mutation (Leclere et al., 2018) or 9-codon deletion in an auxin transcriptional repressor (Figueiredo et al., 2021), or rapid necrosis (De Queiroz et al., 2020) have also been reported. These recent findings highlight that herbicide selection for many survival mechanisms will occur and increase the chances for plants to harbor multiple resistance mechanisms. Multiple herbicide resistance highlights the concurrent dynamic spread of multiple resistance alleles in weeds which exposes a serious threat to productivity of current cropping systems.

## Recent Advances in NTSR Mechanisms

Mechanisms that can contribute to NTSR are complex and involve several different gene types and families. This molecular and genetic complexity makes the identification of particular genes involved in NTSR difficult. Recent advances in this area have been the identification of putative NTSR genes contributing to enhanced herbicide metabolism (EHM).

The latest finding has been the elucidation for the first time that up-regulation of the AKR enzyme contributes to glyphosate resistance in *Echinochloa colona*, by degrading glyphosate to its metabolite, aminomethylphosphonic acid (AMPA; Pan et al., 2019). This discovery further supports results published in this Research Topic, showing glyphosate metabolism in an *E. crus-galli* population from Portugal (Vázquez-García, Rojano-Delgado et al.). The identification of CYP-450 genes (phase I) that can degrade herbicides from different sites of action (SoA) has been carried out recently. CYP81A subfamily has been shown to metabolize herbicides from at least five chemically unrelated groups, both in *L. rigidum* and *E. phyllopogon* (Dimaano et al., 2020; Han et al., 2021). Unraveling which SoA and chemical herbicide families individual CYPs can metabolize, and their identification in different R species could help predicting metabolic-based cross-resistance patterns and thus assist in chemical options for weed management practices.

CYP-450 has been shown to endow herbicide resistance in broadleaf weed species too, as reported for *Glebionis coronia* to ALS inhibitors in this Research Topic (Hada et al.). It is worth mentioning studies confirming that CYP-450 is involved in 2,4-D metabolism in *A. tuberculatus* (Figueiredo et al., 2018) and *Papaver rhoeas* (Torra et al., 2021). Moreover, in *P. rhoeas*, the same CYP-450 has been shown to confer cross-resistance to both 2,4-D and imazamox in several R populations (Torra et al., 2021).

Phase II herbicide metabolism mainly involves conjugation to GSH mediated by GSH S-transferases (GSTs). Metabolic resistance to VLCFA inhibiting herbicides such as flufenacet and pyroxasulfone in *Alopecurus myosuroides* and *L. rigidum* populations is possible due to enhanced GST-mediated metabolism *via* differentially expressed GSTs (Dücker et al., 2019, 2020; Goggin et al., 2021). In this Research Topic, empirical evidence of herbicide metabolism *via* CYP-450 is provided in three articles (Yanniccari, Gigón et al.; Chen et al.; Hada et al.), of GST in two (Wang et al.; Rangani et al.), and of both CYP-450 and GST in five studies (Scarabel et al.; Shyam et al.; Suzukawa et al.; Franco-Ortega et al.; Torra et al.).

All types of resistance mechanisms can get stacked in R plants, both TSR and NTSR, but also different genes conferring EHM. Several studies have reported over-expression of many genes in NTSR plants compared to S ones, also including those encoding for degrading enzymes such as CYP-450 and GST (Gaines et al., 2020). However, this does not necessarily imply a process of recurrent selection and the concomitant slow accumulation of metabolic resistance genes in a R population. There is evidence that differentially expressed genes responsible for EHM could be under genomic co-expression clusters or across long chromosomal intervals (Giacomini et al., 2020). One major implication of this clustering is the likelihood of a shared mechanism of gene regulation for these regions with NTSR genes. Therefore, potentially, a single gene, that is, a single resistance mechanism, could be responsible of the reported over-expression of several genes involved in EHM and NTSR.

In this Research Topic, Franco-Ortega et al. suggested that plant responses to biotic and abiotic stressors are integrally linked to NTSR-based herbicide resistance mechanisms. Regulation of gene expression involved in stress-response and NTSR is probably a complex process but may include herbicide-responsive genes. Recently, HPPD-inhibiting herbicide responsive genes have been found in *A. tuberculatus*, with little overlap in gene expression patterns between R and S genotypes bringing out dynamic differences in response to herbicide treatment (Kohlhase et al., 2019). Similarly, a contributing article in the present Research Topic, points out that S-metolachlor (VLCFA inhibitor) can further increase the expression of two GSTs in R plants (Rangani et al.).

Differential herbicide translocation between S and R plants constitutes another set of NTSR mechanisms. Membrane carrier proteins (ABC family) are already being unveiled and suggested to be involved in phase III of EHM (Gaines et al., 2020). Although reduced glyphosate translocation was described as a resistance mechanism long ago, only recently the first glyphosate cell membrane carrier has been identified (ABCC-type transporter) conferring glyphosate resistance in *E. colona* (Pan et al., 2021). Active root exudation as a NTSR mechanism has been recently reviewed by Ghanizadeh and Harrington (2020). This mechanism could contribute to imazamox resistance in *Euphorbia heterophylla* (Rojano-Delgado et al., 2019) and MCPA resistance in *Raphanus raphanistrum* (Jugulam et al., 2013).

### Rapid Necrosis: An Intriguing Mechanism of Herbicide Resistance

A fast and localized effect of glyphosate and 2,4-D has been identified in *Ambrosia trifida* (Brabham et al., 2011) and *Conyza sumatrensis* (De Queiroz et al., 2020). This phenomenon has been called rapid necrosis (RN), and was primarily proposed as Phoenix resistance (Gressel, 2009) as apparent “dead” plants were able to regrow a few days after herbicide treatment. The physiological basis of this surviving mechanism is unknown and thus, the classification of RN as TSR or NTSR is difficult. The RN caused by 2,4-D may be related to defective Aux/IAA repressors, TIR1/AFB receptors and ARF transcription factors and in that case would be classified as TSR since these proteins are directly related to the 2,4-D action. Exogenous application of aromatic amino acids decreased RN in *A. trifida* caused by glyphosate (Moretti et al., 2018), indicating a potential TSR mechanism of resistance.

In both 2,4-D and glyphosate cases, a potential reduced herbicide translocation resistance mechanism could be related to ABC transporters (Pan et al., 2021), however, alterations in translocation and cell exclusion resulting in 2,4-D and glyphosate resistance were not identified with the RN phenotype. Some evidence suggests that programmed cell death may be caused not only by pathogens as originally discovered but also triggered by other biotic and abiotic stresses such as herbicides (Burke et al., 2020). Several studies have reported the influence of environmental effects on the occurrence and variability of RN (Harre et al., 2018; De Queiroz et al., 2020), which highlight the difficulties of studying RN under the variable conditions found in the field and experimental conditions. Distinguishing the biochemical processes that cause RN from those that are the consequence of RN is needed to better understand this intriguing herbicide resistance mechanism.

### Contributions in the Research Topic

Contributions in this Research Topic reported both TSR and NTSR mechanisms. Eight out of 13 articles reported mechanisms of TSR nature (all substitutional mutations), which in some cases can confer cross-resistance to different herbicide chemistries within the same SoA (Scarabel et al.; Vázquez-García, Alcántara-De La Cruz et al.; Yanniccari, Gigón et al.; Hada et al.; Torra et al.). Among these contributions, we shall highlight those reporting multiple-resistance through the accumulation of several substitutional point mutations in different herbicide target enzymes involving ALS, ACCase and EPSPS inhibitors (Scarabel et al.; Vázquez-García, Alcántara-De La Cruz et al.).

In relation to NTSR mechanisms, three contributions reported about herbicide differential absorption and translocation (Suzukawa et al.; Vázquez-García, Rojano-Delgado et al.; Yanniccari, Vázquez-García et al.), whereas most of them (11 out 13) documented cross-resistance due to some level of EHM. It is also remarkable that seven contributions demonstrated the co-evolution of TSR and NTSR mechanisms at both plant and population level.

Resistance to ALS, ACCase, and EPSPS inhibiting herbicides are the most reported cases in this Research Topic, with 8, 7, and 5 contributions, respectively, which agrees with the SoA herbicides most related to herbicide resistance worldwide (Heap, 2021). Resistance to pre-emergence herbicides in different cropping systems is reported, as multiple resistance in combination to the three previously mentioned post-emergence SoA herbicides. Resistance to microtubule assembly (Suzukawa et al.; Chen et al.; Franco-Ortega et al.), VLCFA (Suzukawa et al.; Rangani et al.; Torra et al.), PSII (Shyam et al.; Franco-Ortega et al.; Torra et al.), synthetic auxins (Shyam et al.; Suzukawa et al.; Franco-Ortega et al.; Torra et al.), and both PPO and HPPD in a single six-way-resistant Palmer amaranth (*Amaranthus palmeri*) population (Shyam et al.) are contributions in this Research Topic.

Ten out of 13 contributions reported on herbicide resistance in grass weed species, and three in broadleaf weeds. *Lolium* ssp. is the most reported genus in the Research Topic (six articles), followed by *A. myosuroides* and *Bromus* ssp. (2), and *E. crus-galli* and *Beckmannia syzigachne* (1). Among dicots, two articles reported on the global invasive weed species *A. palmeri* (Shyam et al.; Rangani et al.) and one contribution on *Glebionis coronaria* (Hada et al.).

## Current Research Gaps and Prospects

(1) Multiple herbicide resistance may result from co-evolution of both NTRS and TSR mechanisms (Vila-Aiub et al., 2005; Powles and Yu, 2010; Bostamam et al., 2012; Gherekhloo et al., 2017; Peterson et al., 2018; Cao et al., 2021). An intriguing question is the evolutionary and ecological consequences of the interaction between NTSR and TSR mechanisms in protecting single plants from herbicide damage (Raymond et al., 1989). For instance, point resistance mutations co-existing with up-regulation of herbicide metabolism (EHM by CYP-450 or GST), both endowing resistance to herbicides targeting the same SoA are ubiquitous in resistant weeds (Tardif and Powles, 1994; Chen et al., 2020a,b). Do these resistance mechanisms combine their effects on plant protection in an additive or multiplicative mode? Would it be possible for a single mechanism to endow the maximum protection level making the addition of a second mechanism an ecological redundancy?
(2) Improved understanding of the biology of plant systems will benefit the understanding of gene regulation of NTSR and the effects of environmental factors on the evolution of herbicide resistance. Further studies related to epigenetic regulation caused by direct or indirect herbicide effects will further increase our understanding of herbicide resistance. The NTSR mechanisms associated with EHM are dependent on a complex gene regulation and we are currently just discovering the final players of a large network. Advances on CYP-450 and GST gene identification as well as their regulation and crystallographic information will reveal a fascinating environmental-plant-herbicide interaction system.
(3) Current recommendations for pesticide resistance prevention are based on rotation and mixing of different SoA pesticides (Bourguet et al., 2013; Baym et al., 2016). However, rotation and/or mixing of herbicides resulting in a similar selection pressure for a particular resistance mechanism (e.g., EHM) will increase the risk of resistance evolution (Comont et al., 2020). The advances in the knowledge of NTSR mechanisms will be necessary for making resistance management decisions involving the use of herbicides targeting different metabolic networks, assuming it is possible to avoid development of some of these resistance mechanisms by modifying management.

## Author Contributions

All authors conceived and wrote this Editorial contribution for the Research Topic *Multiple Herbicide-Resistant Weeds and Non-target Site Resistance Mechanisms: A Global Challenge for Food Production*.

## Conflict of Interest

The authors declare that the research was conducted in the absence of any commercial or financial relationships that could be construed as a potential conflict of interest.

## Publisher's Note

All claims expressed in this article are solely those of the authors and do not necessarily represent those of their affiliated organizations, or those of the publisher, the editors and the reviewers. Any product that may be evaluated in this article, or claim that may be made by its manufacturer, is not guaranteed or endorsed by the publisher.
